# Combined Ultrasound and Pulsed Electric Fields in Continuous-Flow Industrial Olive-Oil Production

**DOI:** 10.3390/foods11213419

**Published:** 2022-10-28

**Authors:** Giorgio Grillo, Luisa Boffa, Emanuela Calcio Gaudino, Arianna Binello, Duarte Rego, Marcos Pereira, Melchor Martínez, Giancarlo Cravotto

**Affiliations:** 1 Department of Drug Science and Technology, University of Turin, Via P. Giuria 9, 10125 Turin, Italy; 2 Energy Pulse Systems, Est Paco Lumiar Polo Tecnológico Lt3, 1600-546 Lisbon, Portugal; 3 Acesur, Carretera de La Carolina, km 29, Vilches, 23220 Jaén, Spain

**Keywords:** flow ultrasound, pulsed electric field, virgin oil production, polyphenols, tocopherols, coratina, industrial scale

## Abstract

The aim of the present study is to develop a new industrial process for the continuous-flow extraction of virgin olive oil (VOO) using the non-thermal ultrasound (US) and pulsed electric field (PEF) treatments. These technologies have been tested both separately and in combination, with the aim of making the malaxation step unnecessary. The ultrasound-assisted extraction (UAE) and PEF treatments are both effective technologies for VOO production and have been well documented in the literature. The present study combines a new continuous-flow set-up, with four US units and PEF treatment. The industrial-plant prototype is able to improve VOO yields, thanks to powerful non-thermal physical effects (acoustic cavitation and electroporation), from 16.3% up to 18.1%. Moreover, these technologies increased the content of nutritionally relevant minor components, which, in turn, improves VOO quality and its commercial value (overall tocopherols and tocotrienols improved from 271 mg/kg under the conventional process to 314 mg/kg under the US process). The combined UAE and US-PEF process also increased the extraction yield, while overcoming the need for kneading in the malaxation step and saving process water (up to 1512 L per working day). Continuous-flow US and PEF technologies may be a significant innovation for the VOO industry, with benefits both for oil millers and consumers. The VOO obtained via non-thermal continuous-flow production can satisfy the current trend towards healthier nutrient-enriched products.

## 1. Introduction

Virgin olive oil (VOO) is one of the most important commodities in the Mediterranean basin, especially for Italy, Spain and Greece which, based on an EU olive oil farms report [1], collectively represent 73% of total olive oil production and 53% of the total EU 27 olive grove area. According to the Farm Accountancy Data Network (FADN) database, Spain is mainly characterized by olive producers and Greece by olive oil manufacturers, whereas Italy has a combination of olive, olive oil and mixed producers.

Obtained via the physico-mechanical treatment of *Olea europaea* L. fruit without the need for significant heat treatment or the use of solvents, VOO is a high-quality product with particular sensorial features and ascribed functional properties, including antioxidant activity [2]. The formation of olive paste (by means of crushing and malaxing), followed by oil extraction and its separation from wastewater, via decantation, make up the fundamental procedures of olive oil production [3]. In particular, the enzymatic activity after the crushing phase leads to biochemical reactions that give rise to the release of volatile compounds and phenols. For example, the lipoxygenase (LOX) pathway is thought to be responsible for the generation of aldehydes (both saturated and unsaturated), alcohols and esters [4]. The chemical, organoleptic and nutritional characteristics of VOO are therefore a function of the mass transfer of the minor olive compounds and enzymatic activities that occur over the course of the entire oil-production chain [5]. Malaxation is a key step in guaranteeing the quality of the finished product, with this procedure consisting of the slow and continuous mixing of the olive paste with the purpose of breaking the water-oil emulsions formed during olive pressing to thus favor the aggregation of oil droplets [6,7]. As it involves large volumes and small surfaces, malaxation is affected by a highly inefficient heat exchange, making temperature control (lower than 30 °C) essential to guarantee the typical organoleptic characteristics and properties of the obtained oil [8]. The pursuit of high profits and productive yields can drive VOO producers to use more intense malaxation conditions—time, temperature, and oxidative alteration—and this can have a negative effect on the nutritional and sensory qualities of the final product [5].

The importance of innovation in the agro-food industry and the advantages that it can bring in production yields, costs, sensorial features, waste reduction, health concerns and nutritional characteristics is widely recognized [9]. However, very little has changed in industrial olive oil production over last two decades, and this is mainly due to the traditional nature of the product, with consumers typically perceiving tradition as the opposite of innovation [10,11,12]. Nevertheless, we must consider that an enhancement in nutritional values can lead to end users accepting innovative solutions in the agro-food chain. The key roles of knowledge and human capital in determining the competitive growth of VOO production are closely linked to the values of skills creation, technological progress and continuous learning.

Clearly, the development of innovative processes must be directed towards the application of technologies that are able to preserve the particular characteristics and qualities of VOO. Ultrasound-assisted extraction (UAE) is considered to be among the most promising unconventional techniques [13] for use in the food-processing chain [14,15,16]. The possibilities that this easily applicable technology offers in terms of process efficiency and time and cost reduction, together with the preservation and, perhaps, improvement of organoleptic and nutritional characteristics, also makes the application of UAE a good option for olive oil production [8,17,18]. When applied to olive paste, the mechanical effects exerted by a low frequency (20 kHz) ultrasound (US) can be useful in enhancing continuous VOO extraction processes, thus increasing the working efficiency of industrial plants [19,20,21,22,23,24,25]. The cycles of positive and negative pressure occur when US waves propagate in a liquid, giving rise to steam-containing cavities that grow until, not being able to absorb any further energy, they collapse in violent bubble implosions and the well-known phenomenon of cavitation is generated within the fluid itself. Shock waves, liquid microjets, very high temperatures (up to 5000 K) and high-pressure differentials (30–120 MPa) are among the local effects that promote the rupture of the olive fruit cell walls that were not destroyed by the crusher machine, thus freeing, in a few milliseconds, further oil and the minor compounds contained in the intact cells. Furthermore, the coalescence of the lipid droplets occurs as a consequence of the transient pressure effects on the olive paste. Over the last few years, several authors have demonstrated how introducing US into olive oil production can be considered a radical and genuine innovation with the potential to eliminate bottlenecks (e.g., malaxing) in the olive oil production process, thus leading to an efficient and continuous procedure that can resolve the historical controversy between VOO yield increases and quality [20,26]. Studies on the laboratory and semi-industrial scales have demonstrated how UAE enriches VOO antioxidant content (e.g., phenols) without affecting the free acidity or peroxide values, while also giving higher extraction yields and better temperature performance, thus improving its nutritional properties without compromising the sensorial features of the final product [2,6,20]. A combined industrial apparatus, in which a low-frequency US device was coupled with a heat exchanger and microwave apparatus, has very recently been employed to produce VOO with organoleptic properties and LOX behavior, which are comparable to those of traditionally made oil but with improvements in efficiency and product yield [27].

Following the growing demand for innovative food processing, the use of pulsed electric fields (PEF) as an emerging technology alternative to conventional thermal procedures has been extensively evaluated, since it allows unwanted modifications to physical and organoleptic food characteristics to be prevented or reduced [28]. The use of short pulses, from micro- to milliseconds, at high electric fields and with an intensity in the 0.1–30 kV/cm range constitutes the basic principle of this technology. Room temperature conditions or moderated temperatures can be applied. As a consequence of applying an electric field, and thanks to the presence of ions, a flow of electrical current occurs through the treated food, especially when it is liquid or semi-liquid. The PEF effect can lead to the electroporation of cell membranes as the destabilization of the lipid layer increases their permeability. This phenomenon can enhance mass transfer, giving rise to the leakage of small molecules [29,30]. The potential of this technology in relation to the bioactive features of final food products, microorganism inactivation, enhancements in pressing efficacy and juice extraction from vegetal matrices, and the possible shelf-life extension of food products has been particularly stressed [31,32].

Preliminary trials have been performed herein by applying US technology to a cultivar of *Olea europaea*, named Taggiasca, and have shown that the use of US, instead of malaxation, means that there is no need to add water to separate the olive paste in a decanter, thus avoiding considerable water wastage without affecting VOO quality (See Appendix A).

The efficiency of the US and PEF techniques has been evaluated in this work with the aim of developing an innovative system for VOO extraction. In particular, the two technologies have been investigated singularly and in synergic combination on the Taggiasca and Coratina olive cultivars, in accordance with regional and seasonal availability. The first screening was performed on the Taggiasca variety in an US-assisted pilot-scale process (400–800 kg of olives) involving an ultrasonic flow reactor (1 × US) (See Appendix A). Early trial outcomes encouraged the further up-scaling to the industrial scale (1800 kg), which saw four US reactors (4 × US) being connected in series. At this point, the Coratina variety was exploited for oil production due to its extensive availability. Furthermore, a PEF system was combined with 4 × US to evaluate whether the hybrid approach was able to enhance oil quality. Several combined set-ups were tested: 4 × US (alone), PEF (alone), PEF followed by malaxation (PEFM) and a hybrid 4 × US-PEF system (4 × US-PEF). The monitored key parameters used to validate the presented olive oil production approach were extraction yield, organoleptic assessment, phenol, and tocopherol and tocotrienol content as well as an economical and energetic evaluation of the industrial process. The results were discussed and compared with those of the conventional procedure (CTRL), leading to the process intensification accomplished by the enabling technologies in the VOO-extraction procedure being verified.

## 2. Materials and Methods

### 2.1. Olive Cultivars

The Coratina olives were purchased from Frantoio Gandolfo (Imperia, Italy).

### 2.2. Sustainable EVOO Production: Description of the Used Devices

#### 2.2.1. US Devices

**Figure 1:**
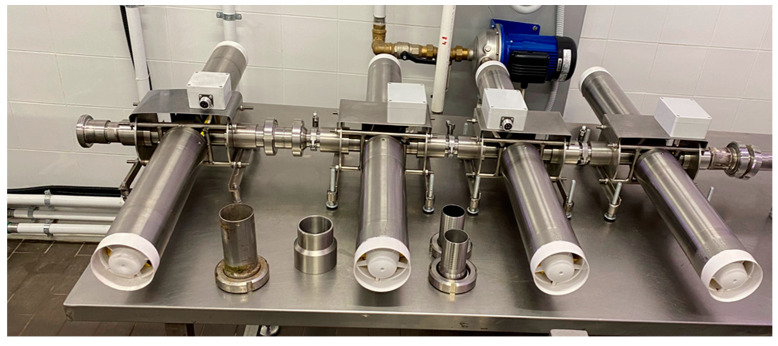
4 × US devices.

The US devices used for the 1 × US, 4 × US and 4 × US-PEF trials contained either one or four ultrasonic flow reactors (internal volume of 330 mL) working at 22 kHz and 600 W, and were equipped with an CAA-GP-1 ultrasonic generator each (maximum power 1000 W) (Figure 1 and Figure S1).

#### 2.2.2. PEF Equipment

**Figure 2:**
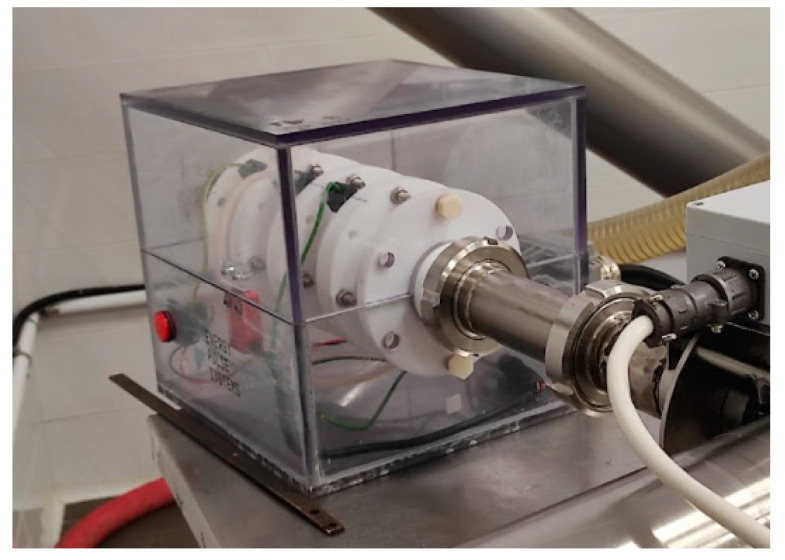
PEF chamber.

The PEF used for the 4 × US-PEF and PEFM trials was an EPULSUS^®^-PM2-15 device (maximum voltage 15 kV; maximum power 6 kW, EnergyPulse Systems, Lisboa, Portugal) (Table 1). The PEF treatment chamber was a DN50 with a 50 mm gap between the electrodes (Figure 2).

In the PEFM trial, the instrument worked with a higher electric field and at a specific energy (2 kV/cm and 5 kJ/kg).

### 2.3. General Procedure for VOO Production

Conventional facilities that are not the focus of this work, such as the hammer crusher and downstream systems (malaxer, decanter and vertical centrifuge), are reported in Table S1.

#### 2.3.1. US-Assisted Industrial-Scale Process (Harvest of Coratina Cultivar 2021)

**Figure 3:**
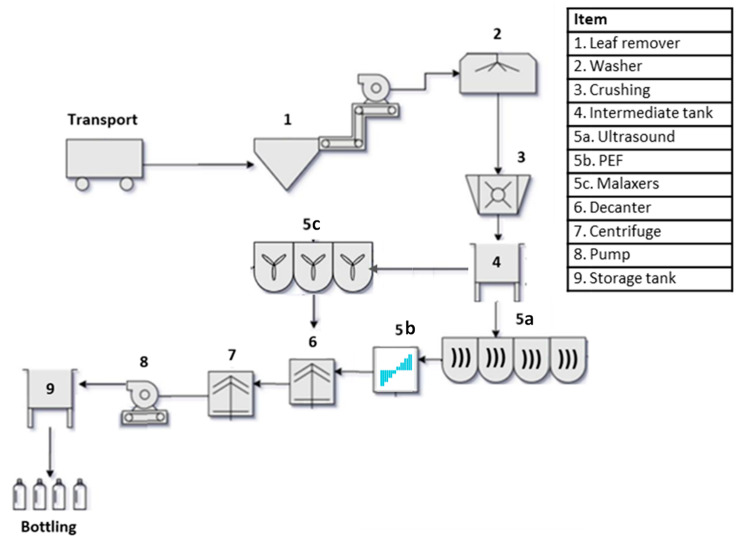
Flow diagram of the 2021 US-assisted industrial process, according to the adopted set-up: 5a: 4 × US; 5a + 5b: 4 × US-PEF; 5b: PEF; 5b + 5c: PEFM; 5c: CTRL.

The US-assisted industrial-scale process trials were performed as follows: 1800 kg of Coratina olives (half-ripe, harvest 2021) were used for the trials and the adopted olive paste flow rate was 900 kg/h. A sonication treatment was performed on the olive paste, instead of the conventional malaxation step, using four US devices operating in sequence (600 W and 22 kHz) (Figure 1). The olive paste was sonicated through the four US devices at a flow rate of 900 kg/h and directly pumped into the decanter. The “olive paste” residence time in each US unit was 3.2 s, and only for that time did the olive paste reach 27–29 °C. No water was added to the sonicated olive paste during the separation phase in the decanter. The process flow diagram, with respective operations, is shown in Figure 3 (5a set-up).

#### 2.3.2. Hybrid 4 × US-PEF-Assisted Industrial-Scale Process (Harvest of Coratina Cultivar 2021)

The hybrid 4 × US-PEF-assisted industrial-scale process trials (harvest 2021) were performed as reported in Section 2.3.1, with the simple addition of a PEF modulus after the 4 × US devices (see Section 2.2.2), working with the parameters reported in Table 1 (see Figure 3, set-up 5a + 5b).

#### 2.3.3. PEF- and PEFM-Assisted Industrial-Scale Process (Harvest of Coratina Cultivar 2021)

The PEF-assisted industrial-scale process trials (harvest 2021) were performed as follows: 1800 kg of Coratina olives (half-ripe) were used for the trials, and the adopted olive paste flow rate was 900 kg/h. No water was added to the olive paste during the separation phase in the decanter.

The process flow diagram, with respective operations, is shown in Figure 3 (5b set-up). In the case of the PEFM-assisted industrial-scale process (harvest 2021), a malaxation step was performed between the PEF and the decanting unit (malaxing step performed for 45 min at 30 °C). The process flow diagram, with respective operations, is shown in Figure 3 (5b + 5c set-up).

#### 2.3.4. Control Procedure for VOO Production (Harvest of Coratina Cultivar 2021)

The CTRL industrial-scale process trials (harvest 2021) were performed as follows: 1800 kg of Coratina olives (half-ripe) were used for the trials, the adopted olive-paste flow rate was 900 kg/h, a malaxation step (45 min at 30 °C) was used and water was added to the olive paste during the separation phase in the decanter. The process flow diagram, with respective operations, is shown in Figure 3 (5c set-up).

### 2.4. General Analytical Procedures for Coratina VOO Analysis

The content and composition of tocopherols, tocotrienols (ISO9936:2016 (E)) and polyphenols [33] and an organoleptic assessment (Reg CEE 2568/1991 11/07/1991 GU CEE L248 05/09/1991 All XII Reg UE 1348/2013 16/12/2013 GU UE L338 17/12/2013 All V Reg UE 1227/2016 27/07/2016 GU UE L202/7 28/07/2016 All II Reg UE 1604/2019 27/09/2019 GU UE L250, 30/09/2019 All IV) for all the Coratina oil samples were determined in an external analytical laboratory (INNOVHUB, Stazioni sperimentali per l’industria; SSOG Stazione sperimentale degli oli e dei grassi; Milan, Italy). Results were expressed as means ± expanded measurement uncertainty with a coverage factor k = 2 and a confidence level of 95%. The analyses were performed according to the official IUPAC method (1992) [34]. A 0.5:99.5 propan-2-ol/hexane mixture was used as the mobile phase and the monitored wavelength was 292 nm.

## 3. Results and Discussion

### 3.1. Industrial-Scale Processes

#### US-Assisted Industrial-Scale Process Coratina (Harvest 2021)

The larger amount of olives that were to undergo treatment, together with the poor Taggiasca harvesting campaign of 2021, forced us to consider a different cultivar for the industrial-scale processes. The Taggiasca variety is only produced in a limited area in northern Italy (Liguria), and, considering that this study aims to explore the possibility of combining US and PEF on an industrial scale for widespread sustainable oil production, we decided to use the Coratina variety, whose farming is more widespread, ranging from the north to the south of Italy (mainly Puglia, Basilicata, Campania and Calabria).

Four different processes for oil production at the industrial scale (1800 kg scale) from Coratina olives were compared:

1. Conventional (Control extraction, CTRL);
2. Four US-assisted industrial-scale process (4 × US);
3. PEF + Malaxation-assisted industrial-scale process (PEFM);
4. Four US − PEF-assisted industrial-scale process (4 × US-PEF).

Schematic representations of the processes that were compared during the 2021 trials (CTRL, 4 × US, 4 × US-PEF and PEFM) are reported in Figure 3 of Section 2.3.1. As can be observed, the malaxation time required for the CTRL (45 min) was completely avoided during the 4 × US process, dramatically cutting down the process time for every kg of oil produced. In addition, the addition of water, 0.21 L/kg required during the CTRL process, for decantation was not required after the olive paste sonication. In general, the non-conventional techniques applied in the trials increased the oil extraction from the half-ripe Coratina olives (Table 2).

**Figure 4:**
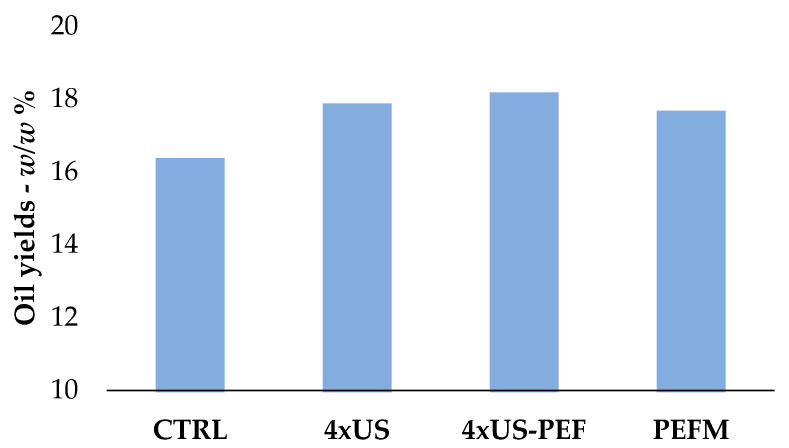
Oil yields obtained from half-ripe Coratina olives using different extraction techniques.

The CTRL in the oil mill gave a 16.3% yield of olive oil, while the US alone (4 × US) and the PEF combined method (4 × US-PEF) provided the highest yield values (near 18%) with an increase of 9.2% and 11%, respectively, over the control. PEF followed by malaxation (PEFM) also increased the oil extraction by 8% over the CTRL (Figure 4).

### 3.2. Oil Analysis

**Figure 5:**
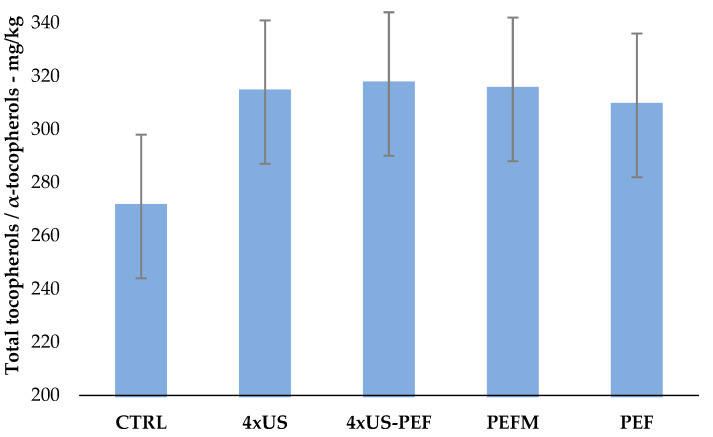
Total tocopherol and tocotrienol content in oil samples obtained from half-ripe Coratina olives according to processing technology (mg/kg) (mean ± U, which represents the expanded measurement uncertainty with a coverage factor k = 2 and a confidence level of 95%).

Sensory evaluation assigned a category of virgin olive oil (VOO) to all the oils produced, since defects were present. The median of the negative attribute with the highest intensity (Md) was between 1.2 and 2, and the defects perceived with the highest intensity were winey and brine. Starting olives had been harvested a few days earlier, influencing the organoleptic assessment of the extracted olive oils. However, the median of fruitiness and the medians of the pungent and bitter attributes were high, ranging from 2.8 to 4. Taking the CTRL as the reference, 4 × US and PEFM oil samples were the best and the most similar, with the highest values in positive attributes and lowest values in negative attributes. On the other hand, the 4 × US-PEF oil sample was the worst, showing the highest value in the negative attributes and the lowest value in the positive attributes. Total tocopherols and tocotrienols were significantly increased by the non-conventional techniques, with similar values for 4 × US, 4 × US-PEF and PEFM (between 314 and 317 mg/kg, increase of 16–17% over CTRL, Figure 5) being observed.

**Figure 6:**
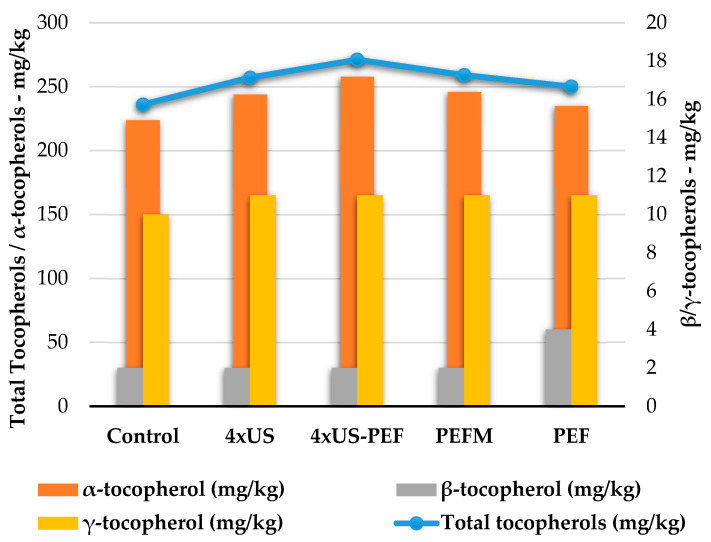
Tocopherol content in oil samples obtained from half-ripe Coratina olives according to processing technology (mg/kg).

In particular, the amount of α-tocopherol (orange bars in Figure 6, axis scale on the left) was strongly affected by 4 × US-PEF, 4 × US and PEFM, with there being a direct influence on the total amount of tocopherols in the collected oils (blue line, axis scale on the left), while β- and γ-tocopherols (yellow and grey bars, axis scale on the right) were less heavily influenced.

**Figure 7:**
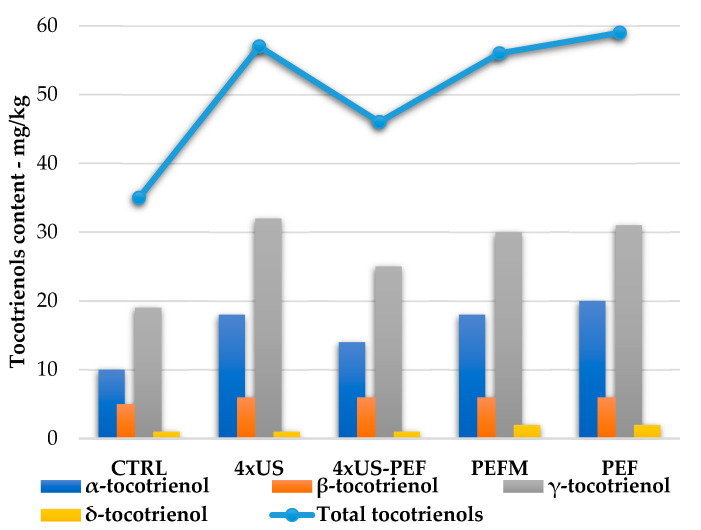
Tocotrienol contents in oil samples obtained from half-ripe Coratina olives according to processing technology (mg/kg).

The amounts of β- and δ-tocotrienols (orange and yellow bars, Figure 7) were not influenced by the applied non-conventional techniques. On the other hand, the contents of γ- and α-tocotrienols (grey and blue bars, Figure 7) were strongly increased by 4 × US-PEF both with and without malaxation, directly influencing the total tocotrienol amount in the oils (blue line, Figure 7).

**Figure 8:**
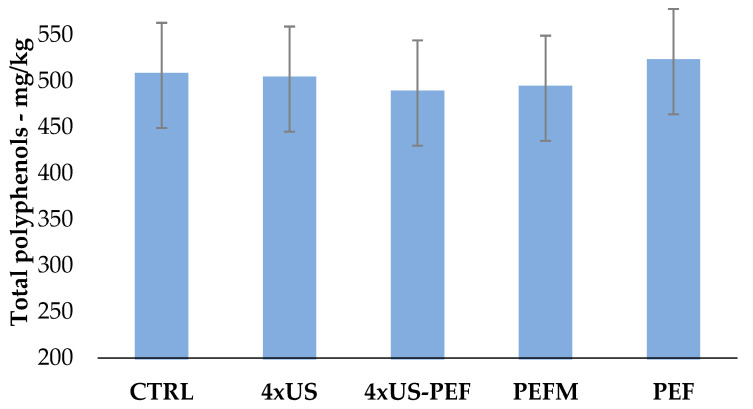
Total polyphenol contents in oil samples obtained from half-ripe Coratina olives according to the processing technology (mg/kg) (mean ± U, which represents the expanded measurement uncertainty with a coverage factor k = 2 and a confidence level of 95%. Results from INNOVHUB laboratory).

In these trials, the total biophenols were not dramatically influenced by the ultrasound and the pulsed electric fields (Figure 8).

Polyphenols in the 4 × US oil were comparable with the CTRL, while the 4 × US-PEF and PEFM values were slightly lower than the CTRL. The PEF sample (without malaxation) had a small increase in polyphenol content.

**Figure 9:**
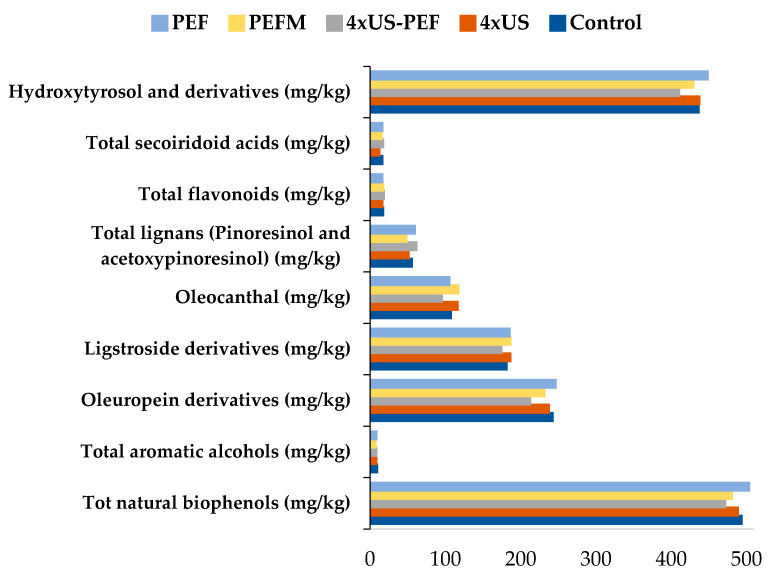
Total biophenols and their composition of Coratina oil samples according to processing technology.

The Coratina VOO samples were rich in oleuropein and ligstroside derivatives, as well as oleocanthal and lignans (pinoresinol and acetoxypinoresinol). The contents of oleuropein, ligstroside derivatives and oleocanthal decreased under the 4 × US-PEF treatment, while lignans slightly increased (Figure 9). A detailed analysis for tocopherols, tocotrienols and polyphenols is reported in Table S2.

### 3.3. Economy and Energy Evaluations of Industrial Processes

The current market requires processes and products with competitive costs, meaning that the industrial sector must invest in technological innovations and tools that evaluate the process economy, making it possible to identify future viability and profitability for producing companies. In this context, we herein report a preliminary economic analysis of the industrial-scale UAE olive oil process (2000 kg olives) and compare it with the manufacturing costs of the conventional extraction process, highlighting how the malaxing step can be avoided.

The estimation of the manufacturing cost (MC) of the UAE of olive oil [35] depends on the five main costs for the installation of all necessary equipment (Equation (1)): a fixed investment capital (FIC), the cost of operational labor (COL), the cost of utilities (CUT), the cost of waste treatment (CWT) and the cost of the raw materials (CRM). All of these parameters are expressed as the unit cost of production (EUR/kg) and are obtained from the relationship between the MC (EUR/year) and the production rate (kg/year).

MC = 0.304 × FIC + 2.73 × COL + 1.23 × (CUT + CWT + CRM) (1)

We herein report the unit costs and energy consumption of both the conventional and UAE processes (Table 3) for the industrial-scale processes that were applied in the 2021 trial. No remarkable differences in total costs (EUR 330,000/340,000) and total energy consumption (~80 KW/h) could be found in the base units for the set-ups. However, the UAE process removes the need for the addition of water when separating the olive paste into the decanter, meaning that less olive mill wastewater (OMWW) will be generated by the UAE process than the conventional one. A water saving of 0.21 L per kg of olive paste treated will be achieved (420 L/h if 2000 kg/h of olive is processed). This will dramatically reduce the waste treatment cost (CWT) for the overall oil-production chain (Equation (1)), making the US-assisted process more competitive and sustainable. Furthermore, the four developed US devices are more compact than the four malaxing units, require less space for installation and can also be vertically stacked.

**Figure 10:**
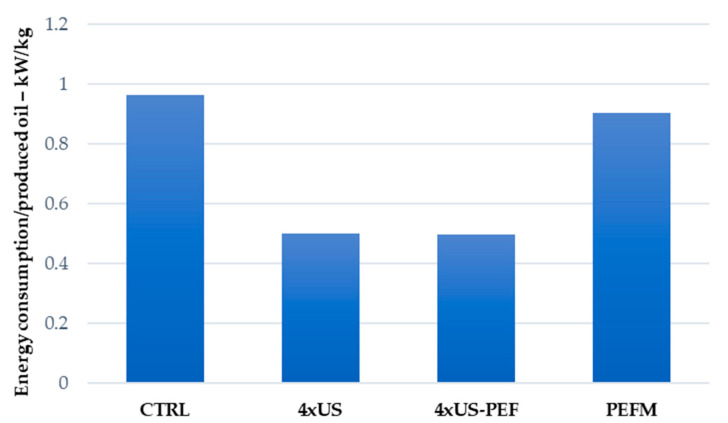
Energy consumption in function of oil yield.

Although the preliminary economic analysis of the 4 × US-assisted process presented herein indicates that it is slightly less expensive (−2.7%) than the conventional process (CTRL) (Table 1 vs. Table 3 and Table 4), we must consider that the US devices herein presented were built specifically for the trials, while large-scale production would certainly have lower production costs. The same evaluation can be made for the combined process, 4 × US-PEF, for which the overall equipment cost was 13.4% higher than that of the CTRL process, due to the additional cost of the PEF unit (EUR 54,500). As shown in Table 5, the 4 × US and 4 × US-PEF trials provided a process flow rate that was almost double that of the CTRL, with 900 kg/h of olive paste being processed to 515 kg/h. These good results, in terms of process rate, were achieved by avoiding the malaxing time (45 min for our pilot scale process). Moreover, the oil yields were higher; the 4 × US process gave a 17.8% yield whereas that of the CTRL was 16.3%, with a daily oil production of 1282 kg/day (4 × US) rather than 672 kg/day (CTRL), calculated over a mean of 8 working hours/day. The same evaluation can be made for the combined 4 × US-PEF process, which provided an 18.1% yield (16.3% CTRL) with a daily oil production of 1303 kg/day instead of 672 kg/day (CTRL). Moreover, the increases in daily oil production provided by the 4 × US and 4 × US-PEF processes also translate into significant energy savings (Table 3 and Table 4). The CTRL process consumes 1.92 times more energy per kg of produced oil than the 4 × US process (0.964 kW/h instead of 0.5 kW/h required by 4 × US process), and 1.93 times more energy than the combined 4 × US-PEF process, as possible to state by direct comparison in Figure 10.

**Figure 11:**
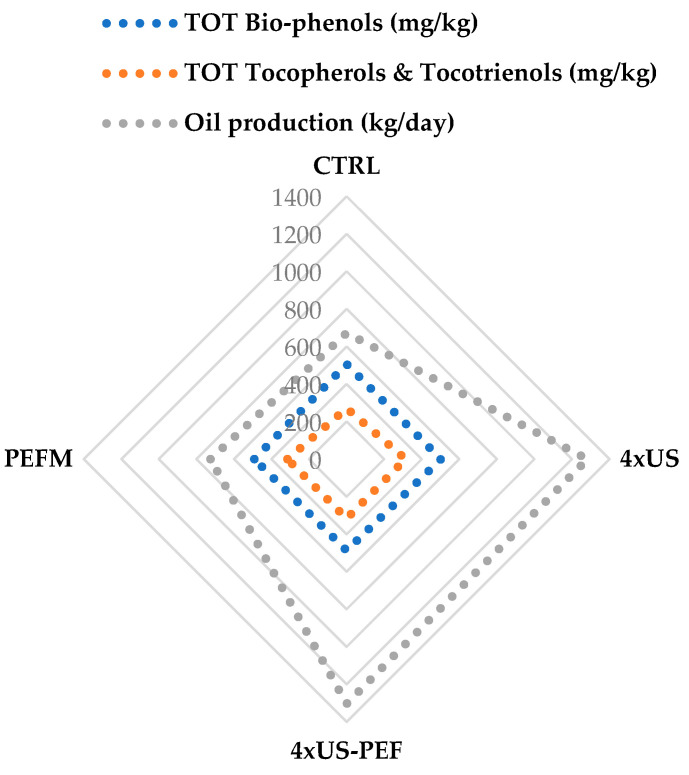
Technology comparison according to overall bio-phenols, tocopherols, tocotrienols and oil production.

In terms of oil quality, a direct comparison between the different technologies is reported in Figure 11 by means of a radar graph. The overall amount of biophenols recorded in the 4 × US process overlapped with that achieved by the CTRL process, while the recorded amounts of total tocopherols and tocotrienols were higher (314 mg/kg) than those achieved during the CTRL process (271 mg/kg) (Table 5). The combined 4 × US-PEF process furnished a small decrease (3.8%) in the total amount of biophenols with respect to the CTRL, while the total amount of tocopherols and tocotrienols was higher than that recorded under both the CTRL (+17.0%) and the 4 × US (+1.1%) processes. Finally, the 4 × US and the 4 × US-PEF processes do not require the addition of 0.21 L water per kg of olive paste treated, lowering the OMWW production and, subsequently, the annual cost for its disposal, which is between EUR 5–15 per ton [36]. When considering 7200 kg of olive paste processed daily (8 h), the consumption of 1512 L of water can be avoided. Previously, Taticchi et al. (2019) [23] presented a high-power US device operating at 20 kHz (2.8 kW power) for the production of olive oil from *Ogliarola garganica* at the industrial scale (2 tons/h). The olive paste coming from the oil mill was processed in a continuous mode with US, but US extraction was always followed by a malaxation phase at 25 °C for 30 min. Although the physical effects of cavitation resulted in a significant increase in the extraction yield at the early ripening stage of the olives (a 22.7% increase compared to the control tests), a small yield increase was observed at the ripe stages (only a 3.4% increase for medium ripening and no documented difference at the advanced ripening stage).

## 4. Conclusions

The present study provides details and results for a new industrial continuous-flow VOO-extraction process under non-thermal US and PEF treatment, alone or in combination. In addition to the VOO yields, the product quality and nutritional properties were also evaluated. Both technologies can improve extraction yield, especially US, with the best results being achieved in the hybrid set-up without malaxation. In detail, an increased oil yield of 17.8% was obtained, with an enrichment in overall tocopherols and tocotrienols of 15.9% and a comparable biophenols concentration (+0.8%). In addition, up to 1512 L of water can be saved in the decanting phase per working day (*ca.* 7200 kg of olives). Lastly, the 4 × US set-up can halve the energy consumed (0.964 kW/kg oil vs. 0.500 kW/kg), mainly due to low running costs and the malaxation step removal.

This is a remarkable result that can increase industrial productivity. Furthermore, it should be stressed that this is not a “plug and play” system; the optimal operating conditions (US and PEF power and parameters) must be tailored to the olive species and maturation level to avoid the risk of secondary-metabolite degradation.

## Figures and Tables

**Table 1:** PEF conditions.

Parameter	4 × US-PEF	PEFM	PEF
Voltage; Pulse; Frequency Electric Field Total Energy Power	[8 kV; 30 µs; 15 Hz] 1.6 kV/cm 3.5 kJ/kg 0.8 kW	[10 kV; 30 µs; 17 Hz] 2.0 kV/cm 5.1 kJ/kg 1.5 kW	[8 kV; 30 µs; 15 Hz] 1.6 kV/cm 3.5 kJ/kg 0.8 kW

**Table 2:** Oil yields for the different extraction processes tested on Coratina olives.

N.	Trial	US	PEF	Malaxing	Oil Yields (% wt)	H_2_O Addition for Decanter
1	CTRL	no	no	yes	16.3	Yes
2	4 × US	yes	no	no	17.8	No
3	4 × US-PEF	yes	yes	no	18.1	No
4	PEFM	no	yes	yes	17.6	Yes
5	PEF	no	yes	no	ND	Yes

ND = not determined.

**Table 3:** Costs of principle equipment and energy consumption.

Item	Base Unit Cost (Euro)	N. of Units	Total Base Cost (Euro)	Energy (kW/h)
1. Leaf remover	19,000	1	19,000	6
2. Washer	23,000	1	23,000	3
3. Crushing	15,000	1	15,000	15
4. Intermediate tank	15,000	1	15,000	0
5a. Ultrasound unit	18,000	4	72,000	3.2
5b. PEF equipment	54,500	1	54,500	0.8
5c. Malaxation container	27,000	3	81,000	4
6. Decanter	95,000	1	95,000	35
7. Centrifuge	35,000	1	35,000	13
8. Pump	17,000	1	17,000	5
9. Storage tank	40,000	1	40,000	0
**Total**			340,000	81

**Table 4:** Industrial trials: results summary.

Trial	Process	Treated Olives	Malax. (Time)	US Flow Rate	Decanter Flow Rate	Process Flow Rate	Daily Processed Olive Paste	Oil Yields	TOT Bio-phenols	TOT Tocols	Oil Prod.	Energy	Energy/ kg Oil
		(kg)	(min)	(kg/h)	(kg/h)	(kg/h) *	(kg/day) **	(% wt)	(mg/kg)	(mg/kg)	(kg/day)	(kW/day) **	(KW/kg)
**1**	**CTRL**	1800	Yes (45 min)	No	900	515	4120	16.3	506	271	672	648	0.964
**2**	**4 × US**	1800	no	Yes (900)	900	900	7200	17.8	502	314	1282	641.6	0.500
**3**	**4 × US-PEF**	1800	No	Yes (900)	900	900	7200	18.1	487	317	1303	648	0.497
**4**	**PEFM**	1800	Yes (45 min)	No	900	515	4120	17.6	492	315	725	654.4	0.903

* Process flow rate calculated considering malaxation time (when required). ** Calculated on 8 h/day working time.

**Table 5:** Industrial trials: energy, costs and production comparison.

Process	Biophenols	Tocopherols and Tocotrienols	Oil Production	Energy Consumption/kg of Produced Oil	Equipment Cost
	(% Reduction) *	(% Increase) *	(% Reduction) *	(% Reduction) *	(Euro, %)
CTRL	-	-	-	-	-
4 × US	0.8	15.9	90.8	48.1	−2.7
4 × US-PEF	3.8	17.0	93.9	48.4	+13.4
PEFM	2.9	16.2	7.9	6.3	+11.6

* The (%) were compared to CTRL data.

**Figure A1:**
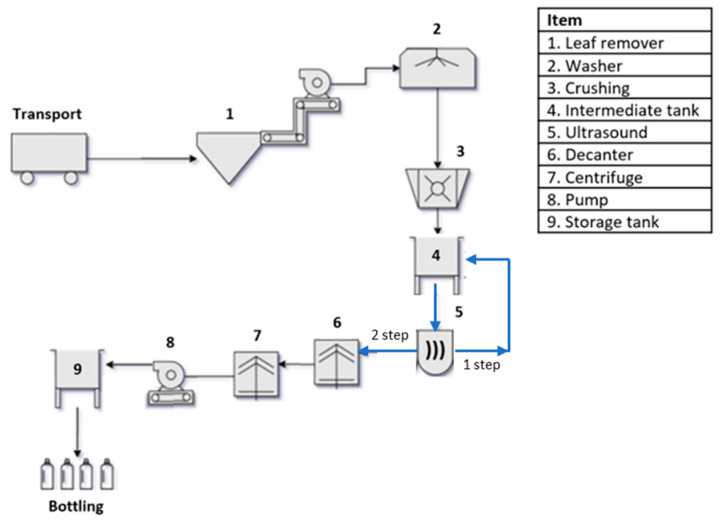
Flow diagram of the 2020 US-assisted pilot-scale process.

**Figure A2:**
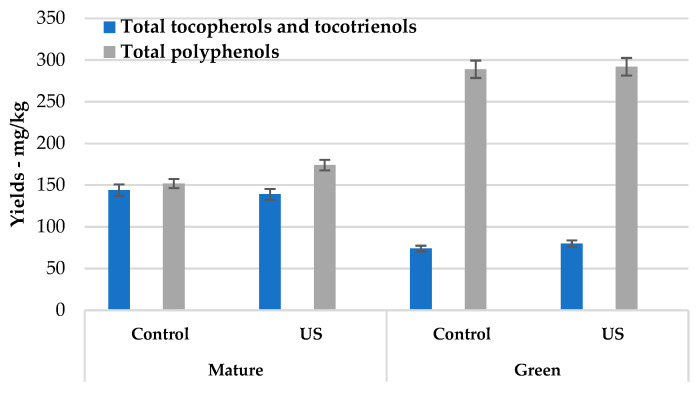
Total tocopherol and tocotrienol (almost α-tocopherol only) and polyphenol contents according to Taggiasca ripening and processing technology (mean ± SE bar).

**Figure A3:**
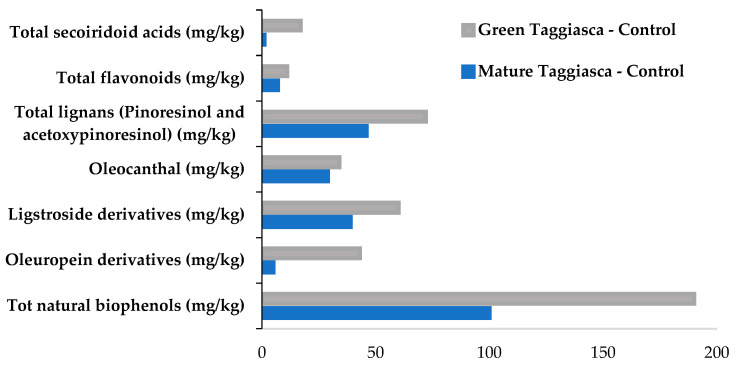
Total biophenols and their composition according to Taggiasca ripening.

## Data Availability

The datasets generated for this study are available on request to the corresponding author.

## Supplementary Materials

The following supporting information can be downloaded at: https://www.mdpi.com/article/10.3390/foods11213419/s1, Table S1: Olive oil mill plant set-up: Conventional facilities.; Figure S1: 2021 4 × US-PEF assisted process.; Table S2: Analysis of olive oils produced by classical oil mill (CONTROL) and by the application of non-conventional techniques (4 × US, 4 × US-PEF and PEF) from half-ripening Coratina variety; Table S3: Analyses of EVOOs produced by classical oil mill (CONTROL) and by the application of non-conventional techniques (ULTRASOUND) from mature and green Taggiasca variety; Table S4: Determination of tocopherols, tocotrienols and polyphenols content in EVOOs produced by classical oil mill (CONTROL) and by the application of non-conventional techniques (ULTRASOUND) from mature and green Taggiasca.

## Appendix A

### Appendix A.1. General Procedure for US-Assisted Pilot-Scale Process (Harvest of Taggiasca Cultivar 2020)

Previous work was conducted in order to develop a proof of concept for the US application to olive paste treatment on a pilot scale. In detail, the conditions used were: 400–800 kg of olives (Taggiasca variety, harvest 2020, Frantoio Gandolfo, Imperia, Italy), an olive paste flow rate of 800 kg/h, a temperature of 25–29 °C and a sonication treatment of 30 min (power 600 W, frequency 22 kHz) using a single US unit. A second US treatment at 450 W (same frequency) was performed by feeding the decanting unit (approx. 30 min), no water was added to the paste during the separation phase. The unit operations of the process were based on a pilot processing plant. The process flow diagram, with respective operations, is shown in Figure A1.

### Appendix A.2. Analysis of Taggiasca Oil Samples

Polyphenol HPLC analyses were performed on a Waters binary pump 1525 linked to a 2998 PDA (Waters Corp., Milford, CT, USA), using a Luna RP C18 column (250 mm, 4.6 mm, 5 µm; Phenomenex, Torrance, CA, USA). HPLC grade solvents and analytical standards were either purchased from Merck (Rome, Italy) or PhytoLab GmbH & Co. KG (Vestenbergsgreuth, Germany).

The analyses were performed according to the COI method [33], with 0.2% H_3_PO_4_ (A) and 1:1 MeCN/MeOH (B) being used as mobile phases. The monitored wavelength was 280 nm, while three-dimensional data were acquired in the 200–400 nm range (gradient: 0 min, 4% B; 40 min, 50% B; 45 min, 60% B; 60 min, 100% B; 70 min, 100% B).

Tocopherol and tocotrienol HPLC analyses were performed on a Waters binary pump 1525 linked to a 2998 PDA (Waters) and used a Spherisorb 5µ Silica column (250 mm, 4 mm, 5 µm; Waters). (±)-α-Tocopherol, rac-β-tocopherol, (+)-γ-Tocopherol and (+)-δ-tocopherol were purchased from Merck.

2-propanol (hypergrade LC-MS LiChrosolv^®^) was purchased from Merck, while n-Hexane (HPLC Grade, 95% min) was obtained from Alfa Aesar (Haverhill, MA, USA). The analyses were performed according to the official IUPAC method (1992) [33]. A 0.5:99.5 propan-2-ol/hexane mixture was used as the mobile phase and the monitored wavelength was 292 nm.

Statistical analyses were performed using R software (R Foundation for Statistical Computing, Vienna, Austria), version 3.6.3 (29 February 2020). The analyses were all performed in triplicate. Residuals were checked for normality using the Shapiro–Wilk and Kolmogorov–Smirnov tests, and variances were checked for homogeneity using Levene’s test.

The results of the total tocopherol and polyphenol contents of the Taggiasca oil samples were processed using the two-way ANOVA statistical technique (two factors: ripening stage and technique, 2 × 2 levels, significant differences and interactions with *p* < 0.05). Tukey’s HSD post-hoc multiple-comparison test was used with a confidence level of 0.95 and a significance level α = 0.05, with the Bonferroni adjustment being applied, when necessary, to determine the differences between the different sample means. When necessary, multiple comparisons were made one factor at a time. The confidence level and p-value adjustments, determined using the Bonferroni method, were based on the number of tests and estimates performed (two, based on the factor considered).

The content and composition of tocopherols, tocotrienols (ISO9936:2016 (E)) and polyphenols (COl/T.20/ Doc. No 29/Rev.1/2017) for Taggiasca CTRL oil samples and an organoleptic assessment (Reg CEE 2568/1991 11/07/1991 GU CEE L248 05/09/1991 All XII Reg UE 1348/2013 16/12/2013 GU UE L338 17/12/2013 All V Reg UE 1227/2016 27/07/2016 GU UE L202/7 28/07/2016 All II Reg UE 1604/2019 27/09/2019 GU UE L250, 30/09/2019 All IV) for all Taggiasca oil samples were determined in an external analytical laboratory (INNOVHUB, Stazioni sperimentali per l’industria; SSOG Stazione sperimentale degli oli e dei grassi; Milan, Italy).

### Appendix A.3. US-Assisted Pilot-Scale Process Taggiasca (Harvest 2020)

US-assisted extraction was performed in a new US reactor, working in continuous mode at 22 kHz and 450–500 W, which provided a direct path for the sonicated product into the decanter without the addition of water. The olive paste was fluid enough after sonication to be pumped and separated into decanters. This sonication step therefore avoided the wastage of 0.21 L of water for each kg of olive paste, resulting in a huge saving of wastewater. This aqueous residue is currently often spread onto agricultural fields in a procedure that is potentially hazardous for the environment (high COD and BOD) and that requires a passive cost of EUR 5–15 per ton. In detail, the water saved during the US-assisted extractive process on Taggiasca olives was 88 L/h. No significant changes were observed in the mass balance of the US and control extractive processes on Taggiasca olives (Table A1); overall oil production was around 10–12% for both the considered processes with this olive variety. The US treatment of olive paste after milling did not negatively affect the total polyphenol content of the final product, but the amount of water required for the further decantation step was reduced. Detailed analysis is reported in Table S3.

**Table A1:** Energy consumption: Control vs. US-assisted extractive process on Taggiasca olives.

Parameters	Mature		Green	
	Control	US ^a^	Control	US ^a^
Process flow rate (kg/h)	~400			
Oil yield (*w*/*w* %)	9	9	11	11
Total Energy Consumption/hour (kW/h)	55	50.8	55	50.8
Energy consumption/kg oil (kW/kg oil)	1.38	1.27	1.14	1.06

(^a^) The US process must be optimized. Indeed, the US device did not work at the theoretical flow of 800 kg/h.

Tocopherol and tocotrienol contents were higher for the mature Taggiasca olives than for the green ones (Table A2, Figure A2). In the two-way ANOVA test, the effect of ripening stage was highly significant for tocopherol content (*p* < 0.001, Table 3, a and b), while the technique employed, and their interaction did not affect it significantly (0.1 < *p* < 1). The analyses showed overlapping values, confirming that the most abundant compound was α-tocopherol (the only compound detectable using the PDA detector). Traces of β- and γ-tocopherol (below 3 mg/kg) and β-, γ- and δ- tocotrienol (below 2 mg/kg) were detected in the oil samples from the mature Taggiasca olives, while only β- and γ-tocopherol (below 2 mg/kg) were observed in the oil samples from green olives. Detailed analysis for tocopherols, tocotrienols and polyphenols is reported in Table S4.

**Table A2:** Total tocopherol and tocotrienol, and polyphenol contents of Taggiasca oil samples (mg/kg).

Compound (mg/kg) (CL, SE) *	Mature		Green	
	Control	US	Control	US
Total tocopherols and tocotrienols (α-tocopherol) (CL 0.95; SE 4.97) ^$^	144 ^a^ (134 ÷ 155)	139 ^a^ (128 ÷ 150)	74 ^b^ (63 ÷ 84)	80 ^b^ (70 ÷ 91)
Total polyphenols (RRF 5.5) (0.95; SE 3.62) ^#^	152 ^c^ (139 ÷ 165)	174 ^e^ (161 ÷ 186)	289 ^d^ (276 ÷ 302)	292 ^d^ (279 ÷ 304)

(*) CL = Confidence level used: 0.95, SE = standard error. (^$^) P-value adjustment: Tukey method for comparing a family of four estimates, significance level used: α= 0.05. (^#^) Conf-level adjustment: Bonferroni method for two estimates, significance level used: α = 0.05. (^a,b^) Tukey post-hoc comparisons on tocopherols and tocotrienols between oil samples. (^c,d,e^) Tukey post-hoc comparisons on polyphenols between oil samples.

Considering both the control and US techniques, significant differences were found in the polyphenol contents in oils obtained from green and mature olives (two-way ANOVA, Tukey’s HSD post-hoc multiple comparison test, one factor at a time, *p* < 0.001). The polyphenol content was higher for the green Taggiasca olives than for the mature olives. Considering the green olives, no significant difference was found between the polyphenol contents in the oils obtained under classical conditions (Control) and under US, while the US increased the polyphenol content for mature olives significantly (Table A2).

The polyphenol composition of the control oil samples of the mature and green Taggiasca olives was evaluated (Figure A3), and higher amounts of secoiridoid acids, lignans, flavonoids and ligstroside and oleuropein derivatives were observed in green olives. In detail, the oleuropein derivatives decreased to 11% of their starting value in the oil samples of mature Taggiasca olives, whereas lignans and flavonoids decreased to 64%, ligstroside derivatives to 65% and secoiridoid acids to 5.9%. The HPLC profiles of the control and US samples did not present any significant differences in polyphenol profile.
