# Supplementary material for: Combined Ultrasound and Pulsed Electric Fields in Continuous-Flow Industrial Olive-Oil Production
Source: Foods. 2022 Oct 28;11(21):3419. doi: 10.3390/foods11213419 (PMC9659190; doi:10.3390/foods11213419)
Supplement: Supplementary file 1 [file foods-11-03419-s001.zip › foods-1965047-supplementary.pdf]

Supplementary Materials

Table S1: Olive oil mill plant set-up: Conventional facilities.

**Hammer Crusher**	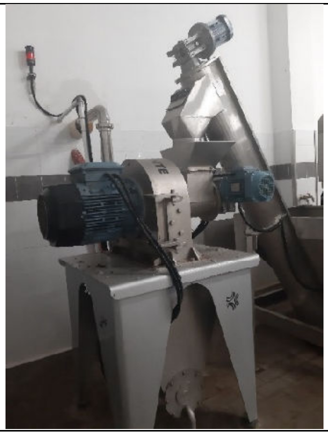	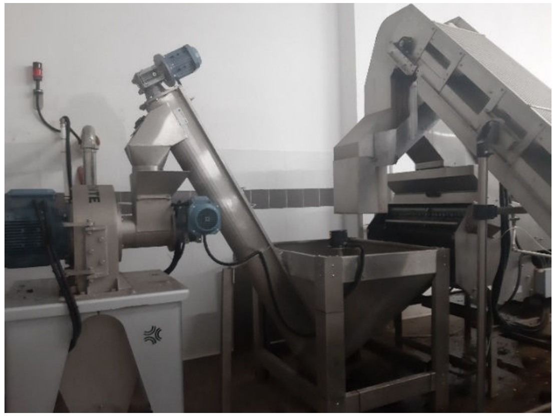
**Malaxer**	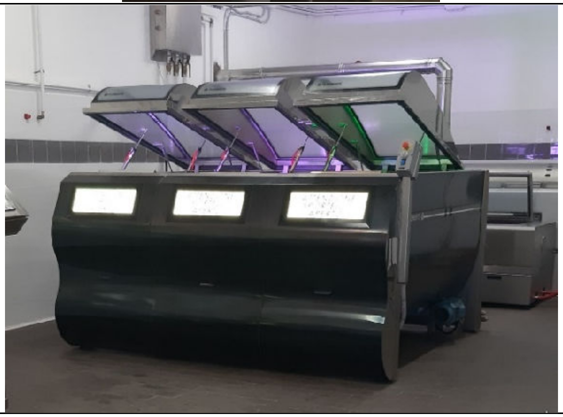	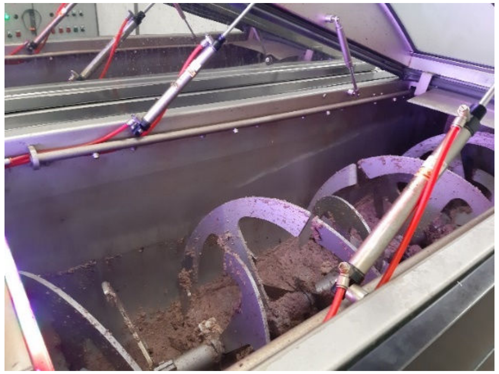

**Extraction** (*3-phase Decanter)*	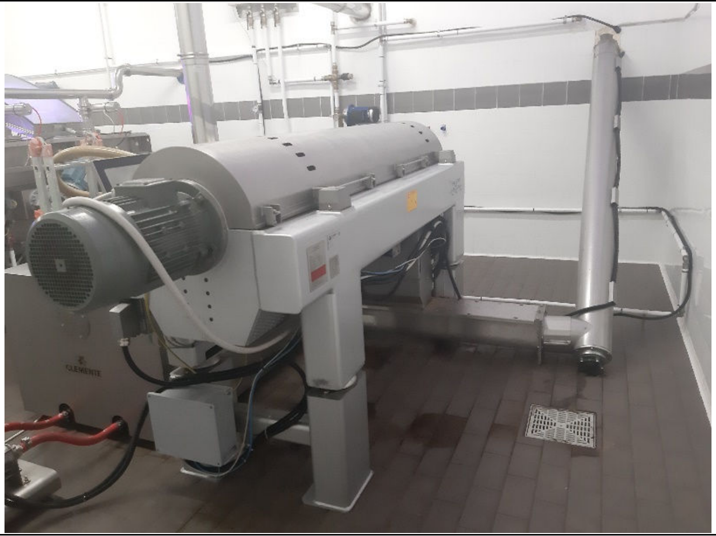	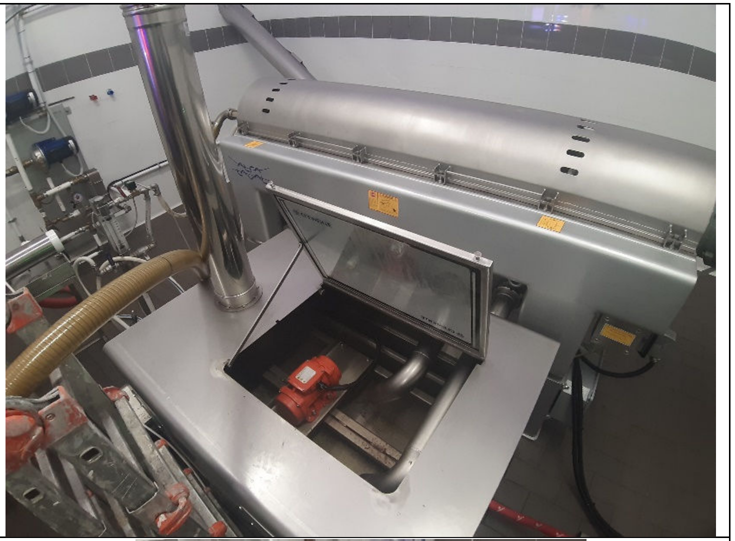
**Extraction** (*Vertical Centrifuge)*	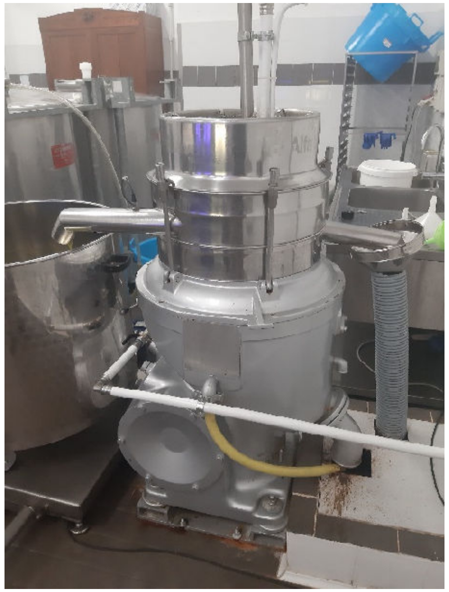	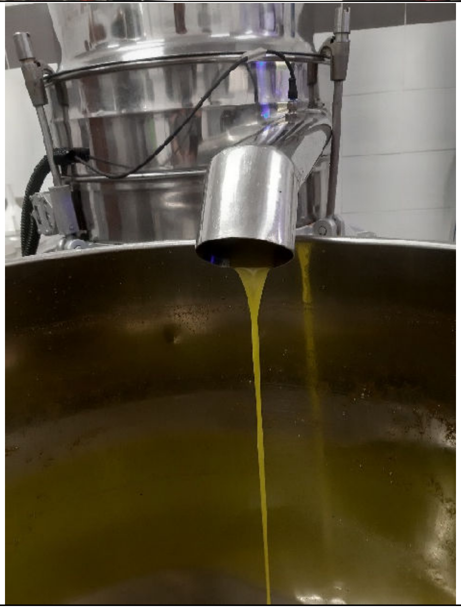

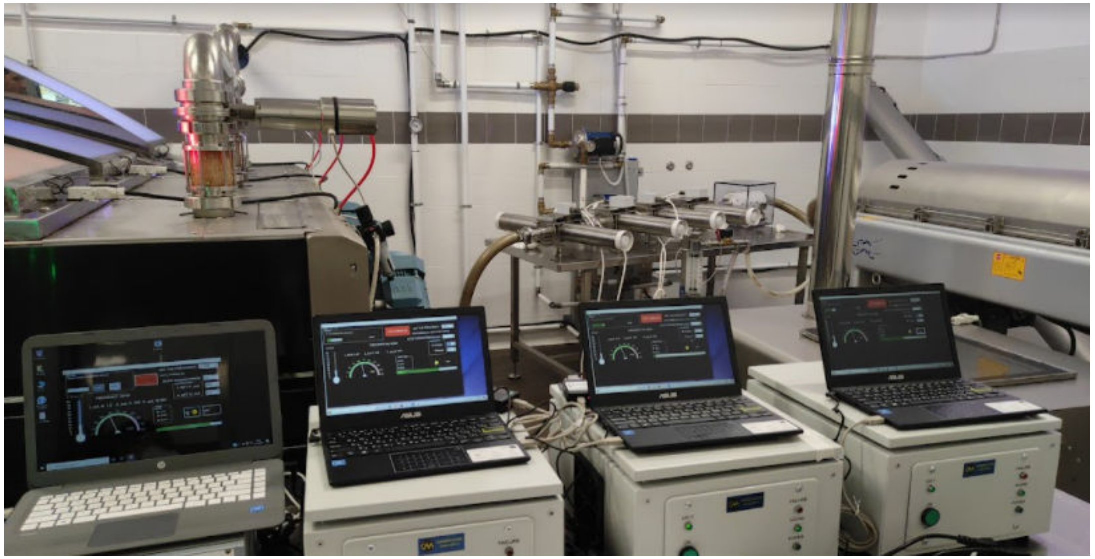

Figure S1: 2021 4xUS-PEF assisted process.

Table S2: Analysis of olive oils produced by classical oil mill (CONTROL) and by the application of non-conventional techniques (4xUS, 4xUS-PEF and PEF) from half ripening **Coratina variety**.

**Analysis**	**Method**	**Compound or test (Meas. Unit)**	**Half ripening Coratina**				
			**Control**	**4xUS**	**4xUS-PEF**	**PEFM**	**PEF**
		α-tocopherol (mg/kg)	224	244	258	246	235

Tocopherols and tocotrienols*	ISO 9936:2016 (E)	β-tocopherol (mg/kg)	2	2	2	2	4
		γ-tocopherol (mg/kg)	10	11	11	11	11
		δ-tocopherol (mg/kg)	<1	<1	<1	<1	<1
		Total tocopherols (mg/kg)	236	257	271	259	250
		α-tocotrienol (mg/kg)	10	18	14	18	20
		β-tocotrienol (mg/kg)	5	6	6	6	6
		γ-tocotrienol (mg/kg)	19	32	25	30	31
		δ-tocotrienol (mg/kg)	1	1	1	2	2
		Total tocotrienols (mg/kg)	35	57	46	56	**59**
		Total tocopherols and tocotrienols (mg/kg ±U)[§]	271	314	317	315	**309**
		Vitamin E (D-α-tocopherol) (mg/100 g)	22	25	25	26	25
		Vitamin E (D,L-α-tocopherol) (mg/100 g)	17	18	19	19	18
Polyphenols*	COI/T.20/ Doc. No 29/Rev.1/2017	Total Biophenols (mg/kg ±U) [§] (RRF (5.2))	506	502	487	492	521
		Tot natural biophenols (mg/kg)	492	487	470	479	502
		Total aromatic alcohols (mg/kg)	8	7	7	6	7
		Hydroxytyrosol (mg/kg)	5	5	5	4	5
		Tyrosol (mg/kg)	3	2	2	2	2
		Oleuropein (mg/kg)	4	4	6	3	4
		Oleuropein derivatives (mg/kg)	241	236	211	230	245

Ligstroside derivatives (mg/kg)	180	185	173	185	184
Oleocanthal (mg/kg)	106	115	94	116	104
Total lignans (Pinoresinol and acetoxypinoresinol) (mg/kg)	54	50	60	47	58
Total phenolic acids (mg/kg)	1	1	1	1	1
Total flavonoids (mg/kg)	16	15	17	16	15
Luteolin (mg/kg)	9	8	10	9	9
Apigenin (mg/kg)	7	7	7	7	6
Total secoiridoid acids (mg/kg)	15	11	16	14	15
Decarboxymethyl elenolic acid (mg/kg)	<1	<1	<1	<1	<1
Elenolic acid (mg/kg)	15	11	16	14	15
Total oxidized biophenols (mg/kg)	14	15	17	13	19
Oxidation ratio % (Total oxidized biophenols/Total biophenols)	2.8	3.0	3.5	2.6	3.6
Hydrolysis ratio % (Total aromatic alcohols/Total biophenols)	1.6	1.4	1.4	1.2	1.3
Total oxidized secoiridoid acids (mg/kg)	<0.1	<0.1	<0.1	<0.1	<0.1
Oxidized decarboxymethyl elenolic acid (mg/kg)	<0.1	<0.1	<0.1	<0.1	<0.1
Oxidized elenolic acid (mg/kg)	<0.1	<0.1	<0.1	<0.1	<0.1
Hydroxytyrosol and derivatives	435	436	409	428	447

(#) REG. CE 2568/91 Annex 1 and Doc. COI/T.15/NC No 3/Rev. 14-2019. (§) U = Expanded measurement uncertainty with a coverage factor k = 2 and a confidence level of 95%. (*) Test not accredited by ACCREDIA.

Table S3: Analyses of EVOOs produced by classical oil mill (CONTROL) and by the application of non-conventional techniques (ULTRASOUND) from mature and green **Taggiasca variety**.

Analysis	Method	Compound or test (Meas. Unit)	EVOO Specification #	Taggiasca variety			
				Mature		Green	
				Control	US	Control	US
Free fatty acids (expressed as oleic acid)	COI/T.20/Doc. No 34/Rev.1 2017	% ±U §	≤ 0.80	0.30±0.07	0.29±0.07	0.37±0.07	0.43±0.07
Peroxide value	COl/T.20/Doc. No 35/Rev.1 2017	meq O_2/kg ±U §	≤ 20.0	7.1±1.1	8.5±2.0	9.9±2.0	10.2±2.0
Insoluble impurities	ISO 663:2017	w/w % ±U §	≤ 0.10	0.01±0.03	0.03±0.03	0.03±0.03	0.01±0.03
Moisture and volatile matter (method B)	ISO 662:2016	w/w % ±U §	≤ 0.20	0.12±0.04	0.16±0.05	0.16±0.05	0.18±0.05
UV spectrophotometric analysis	COl/T.20/Doc. No 19/Rev.5 2019	K232 (CL 0.95; SE 0.033) [a]	≤ 2.50	0.74 (0.62÷0.85)	1.62 (1.50÷1.73)	1.39 (1.27÷1.50)	1.57 (1.45÷1.68)
		K268 (CL 0.95; SE 0.0062) [b]	≤ 0.22	0.09 (0.07÷0.10)	0.09 (0.07÷0.11)	0.10 (0.09÷0.12)	0.11 (0.09÷0.12)
		ΔK (CL 0.95; SE 0.0019) [b]	≤ 0.01	0.00 (0.00÷0.01)	0.00 (-0.01÷0.00)	0.00 (-0.01÷0.00)	0.00 (-0.01÷0.00)
Fatty acids composition	Reg CEE 2568/1991, GU CEE L248 Annex X, Reg UE 1833/2015, GU UE L266/29 Annex IV	Myristic acid (% ±U) §	< 0.03	0.01±0.01	0.01±0.01	0.01±0.01	0.01±0.01
		Pentadecanoic acid (% ±U) §	-	0.01	0.01	< 0.01	0.01
		Palmitic acid (% ±U) §	7.50-20.00	12.08±0.71	12.11±0.71	12.13±0.71	11.97±0.71
		Palmitoleic acid (% ±U) §	0.30-3.50	0.93±0.07	0.93±0.07	0.79±0.07	0.76±0.07
		Heptadecanoic acid (% ±U) §	≤ 0.40	0.05±0.02	0.04±0.02	0.05±0.02	0.05±0.02

		Heptadecenoic acid (% ±U) §	≤ 0.60	0.10±0.02	0.09±0.02	0.10±0.02	0.10±0.02
		Stearic acid (% ±U) §	0.50-5.00	2.12±0.14	2.12±0.14	2.01±0.14	2.00±0.14
		Oleic acid (% ±U) §	55.00-83.00	75.04±0.71	75.05±0.71	75.40±0.71	75.72±0.71
		Linoleic acid (% ±U) §	2.50-21.00	8.05±0.35	8.03±0.35	7.85±0.35	7.66±0.35
		Arachidic acid (% ±U) §	≤ 0.60	0.37±0.07	0.37±0.07	0.39±0.07	0.39±0.07
		Eicosenoic acid (% ±U) §	≤ 0.50	0.33±0.07	0.33±0.07	0.36±0.07	0.37±0.07
		Linolenic acid (% ±U) §	≤ 1.00	0.74±0.07	0.74±0.07	0.72±0.07	0.76±0.07
		Behenic acid (% ±U) §	≤ 0.20	0.12±0.07	0.12±0.07	0.13±0.07	0.13±0.07
		Erucic acid (% ±U) §	-	<0.01	<0.01	<0.01	<0.01
		Lignoceric acid (% ±U) §	≤ 0.20	0.05±0.03	0.05±0.03	0.06±0.03	0.06±0.03
Fatty acids *trans*-isomers	Reg CEE 2568/1991, GU CEE L248 Annex X, Reg UE 1833/2015, GU UE L266/29 Annex IV	Octadecenoic acids (% ±U) §	≤ 0.05	0.01±0.01	< 0.01±0.01	< 0.01±0.01	< 0.01±0.01
		Octadecadienoic + octadecatrienoic acids (% ±U) §	≤ 0.05	0.01±0.01	0.01±0.01	0.01±0.01	0.01±0.01
Sterol composition and content and alcoholic compounds	Reg CEE 2568/1991, GU CEE L248 Annex XIX, Reg UE 1604/2019, GU UE L250 Annex VII	Cholesterol (% ±U) §	≤ 0.5	< 0.1±0.1	0.1±0.1	0.1±0.1	0.2±0.1
		tR Brassicasterol (% ±U) §	≤ 0.1	< 0.1±0.1	< 0.1±0.1	< 0.1±0.1	< 0.1±0.1
		24-Metilencolesterol (% ±U) §	-	0.1±0.1	0.1±0.1	0.1±0.1	0.1±0.1

		Campesterol (% ±U) [§]	≤ 4.0	3.1±0.2	3.1±0.2	3.2±0.2	3.4±0.2
		Campestanol (% ±U) [§]	-	0.2±0.1	0.2±0.1	0.1±0.1	0.2±0.1
		Stigmasterol (% ±U) [§]	< campesterol	0.9±0.1	0.9±0.1	1.3±0.1	1.6±0.1
		Δ-7-campesterol (% ±U) [§]	-	< 0.1±0.1	< 0.1±0.1	< 0.1±0.1	< 0.1±0.1
		Δ-5,23-stigmastadienol (% ±U) [§]	-	< 0.1±0.1	< 0.1±0.1	< 0.1±0.1	< 0.1±0.1
		Chlerosterol (% ±U) [§]	-	0.9±0.1	0.9±0.1	0.9±0.1	0.9±0.1
		β-sitosterol (% ±U) [§]	-	81.6±0.7	81.8±0.7	82.8±0.7	82.5±0.7
		Sitostanol (% ±U) [§]	-	1.2±0.2	1.2±0.2	1.2±0.2	1.4±0.2
		Δ-5-avenasterol (% ±U) [§]	-	10.2±0.1	9.8±0.2	9.0±0.2	8.4±0.2
		Δ-5,24-stigmastadienol (% ±U) [§]	-	1.1±0.1	1.0±0.1	0.7±0.1	0.6±0.1
		Δ-7-stigmastenol (% ±U) [§]	≤ 0.5	0.3±0.1	0.2±0.1	0.2±0.1	0.2±0.1
		Δ-7-avenasterol (% ±U) [§]	-	0.7±0.1	0.7±0.1	0.5±0.1	0.5±0.1
		Apparent β-sitosterol (% ±U) [§]	≥ 93.0	94.7±0.5	94.7±0.5	94.4±0.5	93.9±0.5
		Total sterols (mg/kg)	≥ 1000	1559±123	1552±123	1355±123	1375±123
		Erythrodiol + uvaol (% ±U) [§]	≤ 4.5	1.0±0.6	1.2±0.6	1.1±0.6	0.9±0.6
Tocopherols and tocotrienols	IUPAC 1992	α-tocopherol (the only detectable) (mg/kg) (CL 0.95; SE 7.55) [b]	-	144 (124÷166)	139 (118÷160)	74 (53÷95)	80 (60÷101)

Polyphenols	COl/T.20/ Doc. No 29/Rev.1/2017	mg/kg (CL 0.95; SE 3.62) [a] (RRF 5.5)	-	152 (139÷165)	174 (161÷186)	289 (276÷302)	292 (279÷304)

(#) REG. CE 2568/91 Annex 1 and Doc. COI/T.15/NC No 3/Rev. 14-2019.

(§) U = Expanded measurement uncertainty with a coverage factor k = 2 and a confidence level of 95%.

(a) CL = Confidence level used: 0.95, SE = standard error. Conf-level adjustment: Bonferroni method for 2 estimates, significance level used: $\alpha = 0.05$.

(b) CL = Confidence level used: 0.95, SE = standard error. P value adjustment: Tukey method for comparing a family of 4 estimates, significance level used: $\alpha = 0.05$.

Table S4: Determination of tocopherols and tocotrienols and polyphenols content in EVOOs produced by classical oil mill (CONTROL) and by the application of non-conventional techniques (ULTRASOUND) from mature and green Taggiasca varieties.

**Analysis**	**Method**	**Compound or test (Meas. Unit)**	**Taggiasca**			
			**Mature control**	**US**	**Green control**	**US**
Tocopherols and tocotrienols *	ISO 9936:2016 (E)	Tocopherols (mg/kg)	160	171	108	118
		α-tocopherol (mg/kg)	155	168	105	114
		β-tocopherol (mg/kg)	2	2	1	1
		γ-tocopherol (mg/kg)	3	3	2	2
		δ-tocopherol (mg/kg)	< 1	< 1	< 1	< 1
		Tocotrienols(mg/kg)	4	6	< 1	< 1
		α-tocotrienol (mg/kg)	< 1	< 1	< 1	< 1
		β-tocotrienol (mg/kg)	2	2	< 1	< 1
		γ-tocotrienol (mg/kg)	1	2	< 1	< 1
		δ-tocotrienol (mg/kg)	1	1	< 1	< 1
		Total tocopherols and tocotrienols (mg/kg ±U) [§]	164±27	248±32	108±27	176±34

Biophenols determination in olive oils by HPLC *	COI/T.20/Doc. No 29/Rev.1 2017	Total Biophenols (mg/kg ±U) § (RRF 5.3)	110±41	105±38	207±53	205±52
		Tot natural biophenols (mg/kg)	100	93	190	188
		Total aromatic alcohols (mg/kg)	1	1	3	3
		Hydroxytyrosol (mg/kg)	< 1	< 1	1	1
		Tyrosol (mg/kg)	1	1	2	1
		Oleuropein (mg/kg)	< 1	< 1	< 1	< 1
		Oleuropein derivatives (mg/kg)	5	4	43	42
		Ligstroside derivatives (mg/kg)	39	38	60	62
		Oleocanthal (mg/kg)	29	30	34	37
		Total lignans (Pinoresinol and acetoxypinoresinol) (mg/kg)	46	40	72	67
		Total phenolic acids (mg/kg)	3	2	4	4
		Total flavonoids (mg/kg)	7	6	11	10
		Luteolin (mg/kg)	5	4	7	6
		Apigenin (mg/kg)	2	2	4	4
		Total secoiridoid acids (mg/kg)	1	1	17	12
		Decarboxymethyl elenolic acid (mg/kg)	< 1	< 1	1	1
		Elenolic acid (mg/kg)	1	1	16	12
		Total oxidized biophenols (mg/kg)	10	13	17	18
		Oxidation ratio % (Total oxidized biophenols/Total biophenols)	9.1	10.2	8.2	9.6
		Hydrolysis ratio % (Total aromatic alcohols/Total biophenols)	0.9	1.1	1.5	1.5
		Total oxidized secoiridoid acids (mg/kg)	0.1	0.1	0.3	0.3

	Oxidized decarboxymethyl elenolic acid (mg/kg)	< 0.1	< 0.1	< 0.1	< 0.1
	Oxidized elenolic acid (mg/kg)	0.1	0.1	0.3	0.3

(§) U = Expanded measurement uncertainty with a coverage factor k = 2 and a confidence level of 95%.

(*) Test not accredited by ACCREDIA.
